# Altered Expression of Zonula occludens-1 Affects Cardiac Na^+^ Channels and Increases Susceptibility to Ventricular Arrhythmias

**DOI:** 10.3390/cells11040665

**Published:** 2022-02-14

**Authors:** Mona El Refaey, Sara Coles, Hassan Musa, Tyler L. Stevens, Michael J. Wallace, Nathaniel P. Murphy, Steve Antwi-Boasiako, Lindsay J. Young, Heather R. Manring, Jerry Curran, Michael A. Makara, Kelli Sas, Mei Han, Sara N. Koenig, Michel Skaf, Crystal F. Kline, Paul M. L. Janssen, Federica Accornero, Maegen A. Borzok, Peter J. Mohler

**Affiliations:** 1 Frick Center for Heart Failure and Arrhythmia Research, The Dorothy M. Davis Heart and Lung Research Institute, The Ohio State University Wexner Medical Center, Columbus, OH 43215, USA; hassan.musa@osumc.edu (H.M.); tyler.stevens@osumc.edu (T.L.S.); michael.wallace@osumc.edu (M.J.W.); steve.antwi-boasiako.med@dartmouth.edu (S.A.-B.); young2lj@mail.uc.edu (L.J.Y.); jcurran@abiomed.com (J.C.); makara.5@buckeyemail.osu.edu (M.A.M.); kelli.sas@osumc.edu (K.S.); mei.han@osumc.edu (M.H.); sara.koenig@osumc.edu (S.N.K.); michel.skaf@mountsinai.org (M.S.); janssen.10@osu.edu (P.M.L.J.); federica.accornero@osumc.edu (F.A.); peter.mohler@osumc.edu (P.J.M.); 2 Department of Surgery, Division of Cardiac Surgery, The Ohio State University College of Medicine and Wexner Medical Center, Columbus, OH 43215, USA; 3 Department of Medicine, Duke University School of Medicine, Durham, NC 27601, USA; sara.coles@duke.edu; 4 Department of Physiology and Cell Biology, The Ohio State University College of Medicine and Wexner Medical Center, Columbus, OH 43215, USA; 5 Department of Medicine, Northwestern Medicine, Chicago, IL 62701, USA; nmurphy@iwu.edu; 6 Comprehensive Cancer Center, The Ohio State University College of Medicine and Wexner Medical Center, Columbus, OH 43215, USA; heather.manring@osumc.edu (H.R.M.); crystal.kline@osumc.edu (C.F.K.); 7 Department of Natural Sciences, Mansfield University of Pennsylvania, Mansfield, PA 17101, USA; mborzok@mansfield.edu; 8 Department of Internal Medicine, Division of Cardiovascular Medicine, The Ohio State University College of Medicine and Wexner Medical Center, Columbus, OH 43215, USA

**Keywords:** heart failure, gap junction, sodium channel (Na_V_1.5), connexin-43, Zonula occludens-1 (ZO-1), late sodium current, calcium channel (Ca_V_1.2)

## Abstract

Zonula occludens-1 (ZO-1) is an intracellular scaffolding protein that orchestrates the anchoring of membrane proteins to the cytoskeleton in epithelial and specialized tissue including the heart. There is clear evidence to support the central role of intracellular auxiliary proteins in arrhythmogenesis and previous studies have found altered ZO-1 expression associated with atrioventricular conduction abnormalities. Here, using human cardiac tissues, we identified all three isoforms of ZO-1, canonical (Transcript Variant 1, *TV1*), CRA_e (Transcript Variant 4, *TV4*), and an additionally expressed (Transcript Variant 3, *TV3*) in non-failing myocardium. To investigate the role of ZO-1 on ventricular arrhythmogenesis, we generated a haploinsufficient ZO-1 mouse model (ZO-1^+/−^). ZO-1^+/−^ mice exhibited dysregulated connexin-43 protein expression and localization at the intercalated disc. While ZO-1^+/−^ mice did not display abnormal cardiac function at baseline, adrenergic challenge resulted in rhythm abnormalities, including premature ventricular contractions and bigeminy. At baseline, ventricular myocytes from the ZO-1^+/−^ mice displayed prolonged action potential duration and spontaneous depolarizations, with ZO-1^+/−^ cells displaying frequent unsolicited (non-paced) diastolic depolarizations leading to spontaneous activity with multiple early afterdepolarizations (EADs). Mechanistically, ZO-1 deficient myocytes displayed a reduction in sodium current density (*I*_Na_) and an increased sensitivity to isoproterenol stimulation. Further, ZO-1 deficient myocytes displayed remodeling in *I*_Ca_ current, likely a compensatory change. Taken together, our data suggest that ZO-1 deficiency results in myocardial substrate susceptible to triggered arrhythmias.

## 1. Introduction

Zonula occludens-1 (ZO-1) is a large scaffolding protein located at the gap junctions (GJ) and adherens junctions (AJ) of cardiomyocytes [1]. Specifically, ZO-1 localizes at the AJ as a binding partner of occludin to maintain cell-cell adhesion and at the GJ as a binding partner of connexin-43 (Cx43) for regulating cell-cell communication [2,3,4,5,6]. These roles prove the essential nature of normal ZO-1 function to the intercalated disc (ID), a region at the myocyte-myocyte junction made up of the AJ, GJ, and the desmosome. Alterations in ZO-1 expression and targeting are associated with human diseases including cancer metastasis, increased vascular permeability during inflammation, and regulation of blood-brain barrier in the setting of traumatic intracerebral injury [7,8,9,10]. ZO-1 expression is altered in heart failure, albeit in a contradictory manner. While several studies have identified a decrease in ZO-1 expression in the ventricular myocardium of patients with diverse etiologies of heart failure [1,11], others have found ZO-1 expression to be increased [12]. In addition, several variants within the ZO-1 encoding gene [tight junction protein 1 gene (*TJP1*)] are associated with development of arrhythmogenic cardiomyopathy [13], further supporting the importance of ZO-1 in proper heart function. 

In animal models, loss of ZO-1 influences atrioventricular (AV) nodal function as mice with tamoxifen-induced cardiomyocyte-specific *Tjp1* deletion show normal atrial and ventricular myocardial conduction while exhibiting slowed AV nodal conduction [14]. Furthermore, cardiomyocyte-specific ZO-1 deletion confirmed its unique role in AV conduction by regulating the expression of the cardiac sodium channel (Na_V_1.5) in AV nodal cells [15]. Here, we define the expression of ZO-1 isoforms in non-failing and failing human myocardium and demonstrate a significant reduction of ZO-1 protein expression in failing vs. non-failing human cardiac tissues. To study the role of ZO-1 in cardiac excitability, we generated and used a haploinsufficient mouse model (ZO-1^+/−^; complete knockout of ZO-1 is embryonic lethal [16]). ZO-1^+/−^ mice displayed changes in connexin-43 protein expression and localization at the ID. Adrenergic stimulation resulted in arrhythmia in ZO-1^+/−^ mice, including premature ventricular contractions and bigeminy. ZO-1^+/−^ myocytes displayed a prolonged action potential duration and spontaneous depolarizations, with ZO-1^+/−^ cells displaying unsolicited (non-paced) diastolic depolarizations leading to spontaneous activity with multiple EADs in patch-clamp experiments. Mechanistically, ZO-1^+/−^ myocytes demonstrated a reduction in peak *I_Na_* current density and an attenuation of Na_V_1.5 immuno-reactive signal at the ID. Importantly, late sodium current recordings from haploinsufficient myocytes displayed an increased sensitivity to isoproterenol stimulation. Finally, we observed an increase in *I*_Ca_ current density in ventricular myocytes, likely a compensatory change due to altered function of other cardiac currents. Taken together, we present evidence that further supports the role of ZO-1 in normal electromechanical coupling of cardiomyocytes, that when absent, can lead to arrhythmogenesis. 

## 2. Materials and Methods

### 2.1. Procurement of Human Heart Samples

The studies on human heart tissue were performed under the guidelines of the Declaration of Helsinki, with oversight by the Institutional Review Boards at The Ohio State University. Failing heart samples were obtained from patients who were diagnosed with end-stage heart failure and receiving a heart transplant at The Ohio State University Wexner Medical Center. Non-failing hearts were obtained in collaboration with the Lifeline of Ohio Organ Procurement program from organ donors without diagnosed heart failure whose hearts were not suitable for transplantation. All samples were de-identified. Hearts were removed from the patients/donors and immediately submersed in ice-cold cardioplegic solution (110 mM NaCl, 16 mM KCl, 16 mM MgCl_2_, 10 mM NaHCO_3_, and 0.5 mM CaCl_2_). Tissues were flash-frozen in liquid nitrogen and stored at −80 °C until use. Biopsies from the free wall of the left ventricle were used in this study.

### 2.2. mRNA Expression Analysis

Reverse Transcription (RT) and PCR was performed using Step One Plus and SYBR Green (Applied Biosystems) according to manufacturer’s instructions. Amplicons include the starting exon of *TJP1* transcript variant *TV1* (canonical; NM_003257.4), *TV3* (NM 001301025.2), and *TV4* (CRA_e; NM_001301026.1). Primers were predicted effective by UCSC in-silico PCR, and transcript specificity was confirmed by BLAST search. Primers used include: human *TV1* (Forward: 5′-GAGACAAGATGTCCGCCAGA 3′, Reverse: 5′-AATCCAGGAGCCCTGTGAAG-3′), human *TV3* (Forward: 5′ CGGGGACAAGATGAAGTACCA-3′, Reverse 5′-CGTTACCCACAGCTTCCTCT-3′) and human *TV4* (Forward: 5′-TGGATTGCCACAGTAAGAGCA-3′, Reverse: 5′-AATCCAGGAGCCCTGTGAAG-3′). Quantified mRNA levels were normalized to HPRT. Products were run out on a 0.7% agarose gel to confirm amplicon band sizes. 

### 2.3. Mouse Model

The *Tjp1* locus is located on chromosome 15 within the C57BL/6J mouse genome. To create the targeted knockdown of *Tjp1*, a LacZ-neo-cassette replaced an intronic region between exons 2 and 3.Single allele knockdown was confirmed by PCR analysis of murine genomic DNA using the following primers: data using genotyping with primers to identify both the canonical allele and the neo-cassette ([neoF] Forward primer GGGATCTCATGCTGGAGTTCTTCG; [*Tjp1*R] Reverse primer GGTCATCACTAGCACTGACACCTGG; [*Tjp1*F] TATGCTGTGTGTTCAGAAGCAAGGC). Mice were backcrossed into a C57Bl/6 line. Both age and sex matched littermates were used throughout the study. Males and females showed no phenotypic difference. All experiments involving animals were approved by the Institutional Animal Care and Use Committee at The Ohio State University.

### 2.4. Quantitative RT-PCR

Real-time PCR was performed as previously described [17]. Briefly, Total RNA from the mouse heart tissues was extracted with TRIzol Reagent (Invitrogen) following manufacturer’s instructions. A measure of 1 μg of total RNA, treated with ezDNase, was used for the first-strand complementary DNA synthesis using SuperScript IV Vilo Master Mix (Thermo-Fisher Scientific: Waltham, MA, USA). The qRT–PCR reactions were performed in triplicate in 96-well optical plate with PowerUp SYBR Green Master Mix (Thermo Fisher Scientific). Primers for *Tjp1* are F: 5′-ACAGCACACAGTGACGCTT-3′ and R: 5′-AACCATTGCAACTCGGTCATT-3′. *Hprt* levels were used as a normalization control. Primers for *Hprt* are F: 5′-TGCTGACCTGCTGGATTACA-3′ and R: 5′-TTATGTCCCCCGTTGACTGA-3′.

### 2.5. Tissue Preparation for Immunoblotting

For immunoblotting analysis, whole heart tissues were harvested from mice around 4.5 months old and placed in ice cold PhosphoSafe homogenization buffer (Millipore Sigma 71296-3: Saint Louis, MO, USA). All tissues were homogenized in buffer at 4 °C. The homogenate was centrifuged at 14000 rpm for 15 min at 4 °C, and the resulting supernatant was collected and quantitated by bicinchoninic acid assay (BCA; Thermo-Scientific) prior to analysis. 

For human samples, failing and non-failing cardiac lysates were prepared using RIPA buffer [150 mM NaCl, 1% NP-40 (Nonidet P40 substitute), 0.5% DOC (deoxycholate D-6750), 0.1% SDS (sodium dodecyl sulfate), 50 mM TRIS (PH = 8)]. Samples were centrifuged and the resulting supernatant was quantified as detailed for mouse tissue.

### 2.6. Immunofluorescence

Ventricular myocytes were isolated from WT and ZO-1^+/−^ hearts as we previously described [18]. Isolated cells were fixed and stored in 100% ethanol at −20 °C. Cells were blocked for 1 h in blocking buffer at room temperature (3% gelatin from cold-water fish skin, 1% DMSO and 0.75% Triton-100 (10%) in PBS). Myocytes were stained in primary antibodies in blocking buffer for 3 h at room temperature. Primary antibodies included Na_V_1.5 (1:100, Covance) [19], Connexin-43 (1:200, Invitrogen 710700), ZO-1 (1:100, Invitrogen 40–2300), Ca_V_1.2 (1:100, Alomone ACC-003), alpha-actinin (1:200, Millipore-Sigma A7732) and N-cadherin (1:200, Invitrogen catalog # 33–3900). Myocytes were then stained with secondary antibodies in blocking buffer for 1.5 h at room temperature. Secondary antibodies included Alexa-conjugated donkey anti-mouse 568 and anti-rabbit 488 (1:400, Invitrogen). Myocytes were imaged using identical confocal settings between genotypes and negative controls (stained with only secondary antibodies) on a LSM 780 confocal microscope (Carl Zeiss).

Frozen sections of WT and ZO-1^+/−^ adult mouse hearts were prepared as previously described [20]. Sections were blocked by fish oil blocking agent (3% gelatin from cold-water fish skin, 0.75% Triton x-100 (10%), 1x PBS, 1% DMSO) for 30 min. Sections were incubated in primary antibodies at 4 °C overnight, washed three times in fish oil blocking agent, and then incubated in secondary antibodies for 2 h at room temperature.

### 2.7. Immunoblotting

Adult mouse whole heart lysates were used in immunoblotting experiments. Samples were prepared with Laemmli buffer and separated on 4–15% precast tris-glycine gel (BioRad) and gel was transferred to nitrocellulose (0.45 um). Protein transfer was confirmed with Ponceau and washed with 1 x Tris Buffered Saline + Tween (0.05%) (TBST), then blocked for 1 h at room temperature in 5% non-fat dry milk in TBST or casein. Membranes were incubated overnight at 4 °C with primary antibody, washed three times in TBST, and incubated in secondary antibody for 2 h at room temperature. Blots were developed with Clarity ECL substrate (Bio-Rad 1705061) according to manufacturer’s instructions. If no bands were visible with Clarity substrate, blots were subsequently developed with SuperSignal West Femto maximum sensitivity substrate (Thermo-Fisher Scientific 34095). Densitometry of immunoblotting bands were quantified using Image J software (NIH). Primary antibodies included Connexin-43 (Abcam ab11370 1:1000), Na_V_1.5 (Covance 1:500) [18], Cav1.2 (Alomone ACC003 1:200), GAPDH (Fitzgerald 10R-G109a, 1:5000), alpha-actinin (Millipore Sigma A7732 1:1000) and ZO-1 (Invitrogen 61–7300 1:1000).

For human samples, 20μg of failing and non-failing cardiac lysates were used and primary antibodies included ZO-1 (Invitrogen 61–7300 1:1000) and GAPDH (Fitzgerald 10R-G109a, 1:5000).

### 2.8. Electrocardiograms and Telemetry

Electrocardiograms (ECGs) were obtained from both anesthetized and conscious mice. For conscious mice, telemeters (DSI, St. Paul MN) were implanted subcutaneously in the abdomen of WT and ZO-1^+/−^ mice (*n* = 3) with leads fixed to soft tissue in the lead II orientation. Telemetry was recorded immediately after surgery to confirm lead placement and repeated at 2 and 5 days after surgery. Baseline recordings were taken for ≥ 30 min and continued for 2 h after carbachol (0.15 mg/kg) (Carbamoylcholine chloride, Millipore Sigma C4382), caffeine (120 mg/kg) (Millipore Sigma C1778), or epinephrine (2 mg/kg) administration via intraperitoneal (IP) route. Data collection was completed with Dataquest ART and analyzed using Ponemah Physiology Platform version 5.2 (DSI, St. Paul MN). For anesthetized mice, 2% isoflurane with oxygen (1.0 L/min) was used for sedation prior to subsurface ECG lead placement. Baseline recordings were completed for ≥20 min and continued for up to 1 h following IP injection of isoproterenol (Isoprenaline hydrochloride, 0.05 mg/kg) (Millipore Sigma I5627). Analysis was completed with LabChart 7 Pro (AD Instruments, Sydney, Australia).

### 2.9. Echocardiograms

Transthoracic echocardiographic imaging was performed on mice lightly anesthetized with isoflurane (1.25% in 1 L/min oxygen). Mice were immobilized on a heated pad during image acquisition. Heart rate (HR) was monitored throughout the imaging procedure and recordings were obtained at heart rates higher than 400 bpm. Long and short axis analyses were conducted. Analysis was conducted following acquisition using at least two non-adjacent contractions. Researchers blinded to genotype performed the image collection and analysis.

### 2.10. Electrophysiology

Action potentials were elicited using square wave pulses (1–2 nA amplitude, 1-2-ms duration), generated by a Axopatch 200B amplifier (Molecular Devices, San Hose, CA, USA), and recorded at room temp with pipette solution containing (in mM) 1 MgCl_2_, 1 EGTA, 150 KCl, 5 HEPES, 5 phosphocreatine, 4.46 K_2_ATP, 2 *b*-hydroxybutyric acid, adjusted to pH 7.2 with KOH, and extracellular solution containing (in mM) 148 NaCl, 0.4 NaH_2_PO_4_, 1 MgCl_2_, 5.5 glucose, 5.4 KCl, 1 CaCl_2_, 15 HEPES, and 1 EGTA, adjusted to pH 7.2 with NaOH. Appropriate corrections for access resistance compensation, as well as for leak and capacitive current subtractions were considered.

Whole cell voltage- and current clamp recordings were carried out in isolated adult mouse ventricular myocytes utilizing standard patch clamp techniques. Recordings were performed using the Axopatch 200B (Molecular Devices, Sunnyvale, CA, USA). Sodium currents (*I_Na_*) were recorded at room temperature (20–22°C) with pipette resistances <2.8 MΩ when filled with pipette filling solution containing (in mM): NaCl (5), CsF (135), EGTA (10), MgATP (5), Hepes (5), pH 7.2. The extracellular bathing solution contained (in mM): NaCl (5), MgCl_2_ (1), CaCl_2_ (1.8), CdCl_2_ (0.1). To assess the *I*_Na_ density, cells were held at –160 mV and stepped to various test potentials from –100 to 30 mV in 5 mV increments, with 200 ms duration pulses and 2800 ms interpulse intervals. Voltage-dependence of inactivation was assessed by holding the cells at −160 mV followed by a 300 ms priming pulse from –140 to –40 mV immediately followed by a test pulse to −40 mV; interpulse interval was 2700 ms. Recovery from inactivation was studied by holding cells at −160 mV and applying two 20 ms test pulses (S1, S2) to −45 mV, separated by increasing increments of 1 ms to a maximum S1-S2 interval of 50 ms. The S1-S1 interval was kept constant at 2000 ms.

L-type calcium currents (*I*_Ca_) were recorded at room temperature (20–22 °C) with pipette resistances <3.0 MΩ. The external solution contained the following (in mmol/liter): TEA-Cl (137); CsCl (5.4); MgCl_2_ (1); CaCl (1); 4-AP (4); HEPES (10); glucose (11) adjusted to pH 7.4 with CsOH. The internal solution contained the following (in mmol/liter): CsCl (120); TEA-Cl: (20) MgCl-6H_2_O; Mg-ATP (5.2); HEPES (10); EGTA (10) adjusted to pH 7.2 with CsOH. For *I_Ca_* measurements, myocytes were held at −50 mV followed by 300-ms depolarizing steps to voltages ranging from −40 to +60 mV in 5-mV increments. Voltage-dependence of inactivation was assessed by holding the cells at –140 mV followed by a 300 ms priming pulse from –60 to +10 mV immediately followed by a test pulse to 0 mV; the interpulse interval was 2700 ms. Recovery from inactivation was studied by holding cells at −70 mV and applying two 500 ms test pulses (S1, S2) to 0 mV, separated by increasing increments of 20 ms to a maximum S1-S2 interval of 800 ms. The S1-S1 interval was kept constant at 5000 ms.

Late sodium current (*I*_Na,L_) was measured by integrating the area of *I*_Na_ at 80% repolarization to the 200 ms endpoint of activation and dividing it by the total integration of the current trace (see schematic in Results section) providing the relative contribution of late and persistent sodium current under each condition. A concentration of 1 uM Isoproterenol was used to elicit *I*_Na_,_L_. Pipette resistance was <2.0 MΩ when filled with solution containing (in mM): NaCl (5), CsF (135), EGTA (10), MgATP (5), HEPES (5), pH 7.2. The extracellular solution contained (in mM): NaCl (20), MgCl2 (1), CaCl2 (1.8), CdCl2 (0.1), glucose (11), CsCl (112.5) and Hepes (20); pH was maintained at 7.4 with CsOH. Only myocytes with membrane resistance greater than 1 GΩ were used. Appropriate whole cell capacitance and series resistance compensation (≥60%) was applied. All analyses were done using Clampfit 10.4 software.

### 2.11. Transmission Electron Microscopy

Hearts from control and ZO-1^+/−^ transgenic mice (*n* = 3) were excised, cut into appropriate size pieces (~<1 mm^3^), and processed for TEM according to standard procedure [21], with minor modifications. Specifically, tissue pieces were immediately fixed by immersion fixation in 2% glutaraldehyde, 4% paraformaldehyde in 0.1 M phosphate buffer pH 7.4 containing 0.1 M sucrose overnight at 4 °C. Samples were washed five times in 0.1 M phosphate buffer pH 7.4 containing 0.1 M sucrose, post-fixed for 2 h in 1% osmium tetroxide in 0.1 M phosphate buffer pH 7.4, washed in distilled water followed by a 1-h en bloc stain with ethanolic 2% uranyl acetate. Samples were dehydrated in a graded ethanol series and then transitioned into acetone. A graded acetone:resin (Eponate 12, Ted Pella, Inc., Redding, CA, USA) series was used for tissue infiltration and subsequently samples were embedded in freshly prepared resin in small tapered, flat embedding molds (Ted Pella, Inc.) orientated such that longitudinal sections could be acquired. Blocks were polymerized overnight at 65 °C. Next, blocks were trimmed, and semi-thin sections (500 nm) were acquired using a glass knife. Blocks were further trimmed, and 80 nm thin sections were cut using a diamond knife (Diatome) on a Leica EM UC6 ultra-microtome. Thin sections were collected on 200 mesh copper grids and stained with 1% uranyl acetate and Reynold’s lead citrate. A FEI Tecnai G2 Biotwin TEM operating at 80 kV was used to capture using an AMT camera. Sample imaging and quantification measurements were performed in a blinded manner. Images for measurements were all collected at a magnification of 14,000×. To assess myocyte integrity, sarcomere length was measured from Z-disk to Z-disk. ID integrity was assessed by measuring ID amplitude as well as tread width and step length. In addition, the distances between membranes of opposing myocytes at adhesion, gap, and desmosomal junctions were measured. The width of gap junctions, desmosomes, and adherens junctions were measured at three locations and the values were averaged. The three locations were chosen by dividing each junction/desmosome into three regions based on the total length of the entire junction/desmosome and then measuring within the middle of each section. This avoided any variation that can occur at the distal ends of the junctions/desmosomes. All width measurements were taken between the membranes forming the junction/desmosome and did not include the width of the membranes on each side. Furthermore, the extension of internal membranes and the diameter of transverse tubules were measured. All internal membranes were classified as fully extended, partially extended, or not extended. All measurements were made using the ImageJ software.

### 2.12. Statistics

Data are presented as mean ± SEM. For APD and electrophysiologic studies, data are displayed as mean ± SD. For the comparison of two groups, we performed unpaired two-tailed student t-test when data passed normality test (Shapiro–Wilk normality test). When data did not pass Shapiro–Wilk normality test, we performed Mann–Whitney U test (non-parametric for unpaired comparison) to compare two groups. For the comparison of greater than two groups in human cardiac tissues, immunoblotting data were normally distributed (passed Shapiro–Wilk normality test), ANOVA test was performed for comparing followed by Tukey’s multiple comparison test. For the comparison of failing cardiac lysates vs. non-failing lysates, data passed normality test and we performed an unpaired two-tailed t-test. For our study, a value of *p* < 0.05 was considered statistically significant.

## 3. Results

### 3.1. Identification and Expression-Profile of Different ZO-1 Isoforms in Human Heart

**Figure 1:**
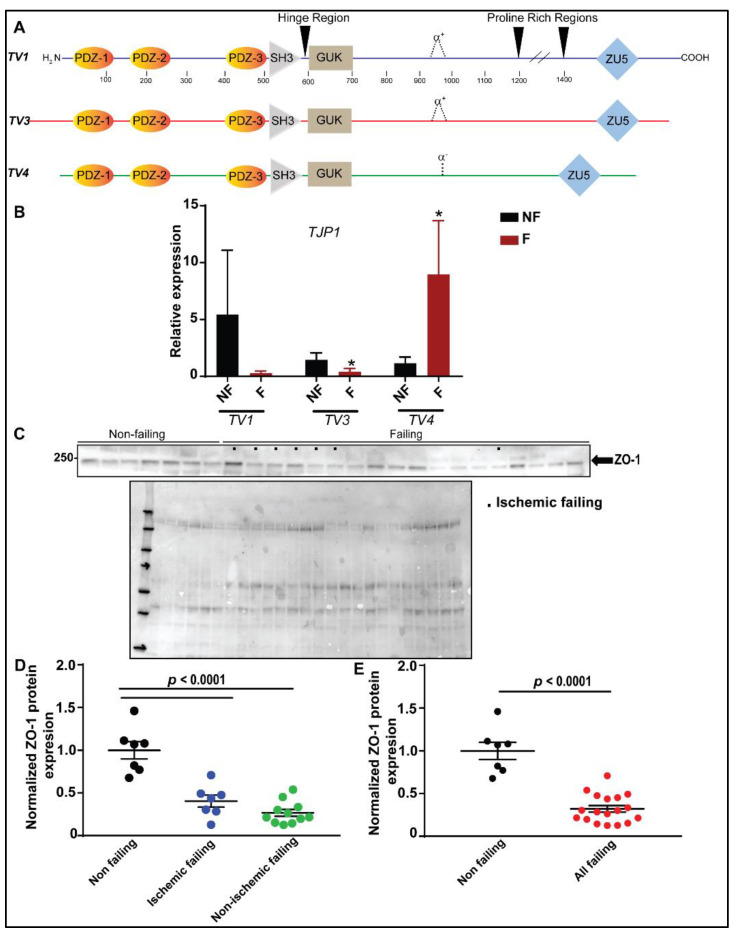
Reduction of ZO-1 protein expression in human tissue samples with diverse etiologies of heart failure. (**A**) Transcript protein maps of the *TJP1* gene. PDZ: Psd95/discs large/zonula occludens-1 domain, SH3: Src homology3 domain, GUK: Guanylate kinase-like domain. (**B**) Expression of *TJP1* isoforms in human non-failing and failing ventricular tissues (*n* = 3 for failing and non-failing tissues * *p* < 0.05) (**C**–**E**) Immunoblotting and quantitative analysis of normalized ZO-1 protein expression (relative to total protein) illustrating reduced ZO-1 protein expression in ischemic and non-ischemic heart failure samples (*n* = 7 for patients with non-failing hearts, *n* = 7 for patients with ischemic failing hearts, *n* = 11 for non-ischemic failing hearts and *n* = 18 for all failing heart samples).

ZO-1 is a tight junction-associated protein that has been found in a variety of cells including epithelial, endothelial and specialized tissue [22]. Willott et al. first described the differential expression of two isoforms of ZO-1, differing by the presence (α+) or absence (α-) of an 80 amino acid motif termed alpha (α), positioned on the C-terminal end of the Guanylate kinase-like domain (GUK) domain [23]. The isoform CRA_e was classified as α- and therefore we expected that it would be found in specialized tissues such as heart and kidney as previously described [24]. To investigate the relative expression of ZO-1 isoforms in human myocardium, we performed qPCR using primers identifying the unique start sites of three isoforms as documented in UCSC of cDNA transcripts of canonical (Transcript Variant 1, *TV1*), CRA_e (Transcript Variant 4, *TV4*), and an additionally expressed (Transcript Variant 3, *TV3*) (Figure 1A).

We identified all three isoforms in non-failing human left ventricle tissues (Figure 1B). Moreover, transcript analysis of *TJP1* gene using qPCR revealed a trending reduction in *TV1* expression in failing human myocardium compared to non-failing. Furthermore, *TV3* expression was significantly decreased and *TV4* expression was significantly increased in failing versus non-failing human cardiac tissues.

Earlier studies on the significance of ZO-1 in heart disease identified a role of ZO-1 in heart failure [1] and atrio-ventricular conduction [15]. Knowing this, we investigated the expression of ZO-1 in tissue samples from individuals with various causes of heart failure. Normalized total ZO-1 protein expression was significantly reduced in both non-ischemic and ischemic failing cardiac tissues compared to non-failing ventricular tissues using a pan ZO-1 antibody recognizing all isoforms described above (Figure 1C–E). Overall, these findings confirm the deficiency of ZO-1 protein expression in patients with diverse etiologies of heart failure.

### 3.2. Reduced ZO-1 Expression Results in Altered Connexin-43 Protein Expression and Localization

**Figure 2:**
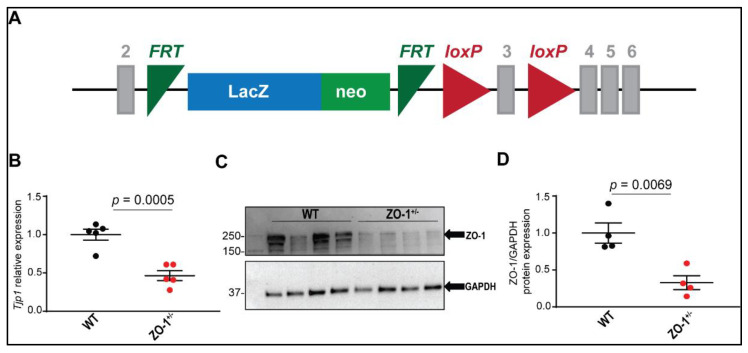
Characterization of ZO-1^+/−^ haploinsufficient mouse model. (**A**) Schematic design illustrating the location of the LacZ-neo-cassette and loxP sites on a single allele of the *Tjp1* gene. (**B**) qPCR data showing a significant reduction of *Tjp1* transcript in ZO-1 deficient mice (*n*=5/genotype and mice were 19 weeks old), WT (two male and three female mice) and ZO-1^+/−^ (one male and four female mice). (**C**,**D**) Immunoblotting and quantitative analysis of heart lysates from control and ZO-1^+/−^ mice confirming a reduction of ZO-1 protein levels in ZO-1 deficient mice (*n* = 4 for each genotype, male mice).

**Figure 3:**
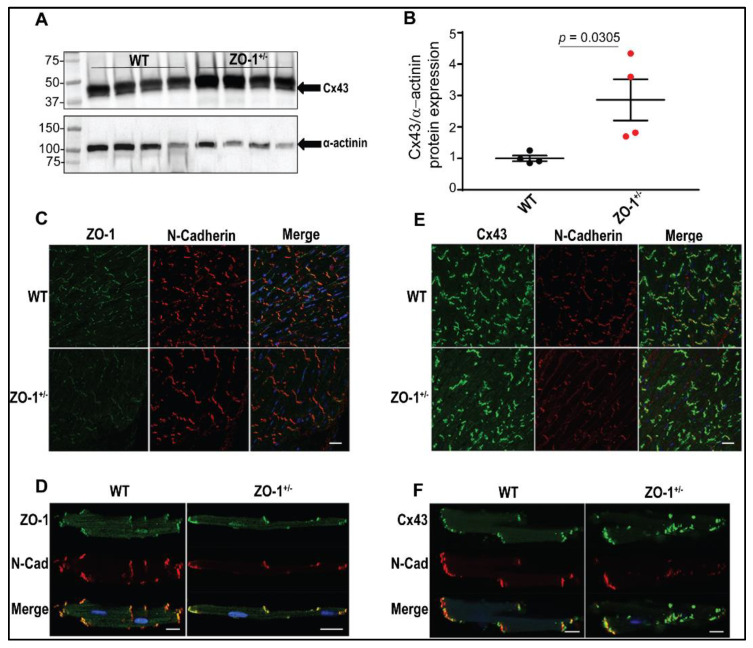
Effect of ZO-1 haploinsufficiency on connexin-43 expression (Cx43) and localization. (**A**,**B**). Immunoblotting and quantitative analysis of Cx43 illustrating a significant upregulation of normalized protein levels in the hearts of ZO-1 haploinsufficient mice (*n* = 4 for each genotype), WT (two male and two female mice) and ZO-1^+/−^ (four male mice). (**C**,**D**) Immunostaining of ZO-1 confirming a reduction of ZO-1 protein expression in the hearts of the ZO-1^+/−^ mice with no changes in subcellular localization within the single isolated ventricular myocytes. (**E**,**F**) Immunostaining denoting an increased expression and an aggregation of Cx43 at the ID of ZO-1^+/−^ myocytes. Scale bars equal 20 μm (**C**,**E**) and 10 μm (**D**,**F**) (*n* = 3/genotype, heart sections: WT (three male mice) and ZO-1^+/−^ (three male mice) and isolated myocytes: WT (one male and two female mice) and ZO-1^+/−^ (three male mice)).

We investigated the consequences of haploinsufficiency of ZO-1 (Figure 2A) on the expression and localization of ID proteins. Quantitative PCR data confirmed a significant reduction in *Tjp1* mRNA levels in ZO-1 haploinsufficient mice (Figure 2B). Using immunoblotting techniques, we confirmed the efficacy of the ZO-1 haploinsufficient mouse model using neo-cassette insertion into a single allele of canonical ZO-1; normalized ZO-1 expression was at ~33% (compared to WT) in ZO-1^+/−^ mouse cardiac lysates (Figure 2C,D). Our data also demonstrated a successful reduction of canonical ZO-1 protein expression at the ID of the ventricular myocytes with no changes in localization at the ID (Figure 3C,D).

Interestingly, we observed an increased expression of connexin-43 in ZO-1 deficient lysates (Figure 3A,B) and an aggregation of connexin-43 at the ID of ZO-1^+/−^ myocytes (Figure 3E,F), that correlates with previous data relating to reduced ZO-1/Cx43 binding and increased gap junction formation at the ID [25]. To further investigate the nature of protein localization in the ventricular myocytes (VMs), we performed Transmission Electron Microscopy (TEM) on ZO-1^+/−^ and WT mice. As shown in (Figure S1), plaques of GJs spanned increased distances between adjacent membranes, averaging 14.6 nm in ZO-1^+/−^ mice versus 11.5 nm in WT mice (Table S1). ZO-1^+/−^ VMs showed a reduction in ID amplitude (0.3 versus 0.4 um), and reduced Step Length (1.2 versus 1.7 um) compared to WT VMs (Table S1). No differences were noted in adherens junction membrane distances, desmosome junction membrane distances, or tread width (Table S1). Internal membrane measurements revealed increased T-tubule axial diameter, specifically in myofilament-myofilament associated T-tubules of ZO-1^+/−^ VMs (90.5 versus 65.7 nm) (Table S1).

### 3.3. Haploinsufficiency of ZO-1 in Mice Does Not Affect Cardiac Function

In view of these data, we considered the consequences of changes in protein expression and localization of ZO-1^+/−^ in the myocardium, and how these might affect cardiac function. To evaluate baseline cardiac function, a group of WT and ZO-1^+/−^, 4–5 months old, were evaluated in terms of cardiac function parameters. Echocardiography demonstrated no statistically significant differences in ejection fraction (EF) (Table S2) or fractional shortening (FS) (Table S2), and therefore no gross differences in cardiac function were noted in ZO-1 haploinsufficient mice at baseline. Additionally, ZO-1^+/−^ mice showed no difference in regular activity such as grooming, social interaction, or eating as compared to WT mice (data not shown). No early or unexplained death was observed in either cohort. 

### 3.4. Adrenergic Stimulation of ZO-1^+/−^ Adult Mice Induces ECG Abnormalities and Arrhythmia

**Figure 4:**
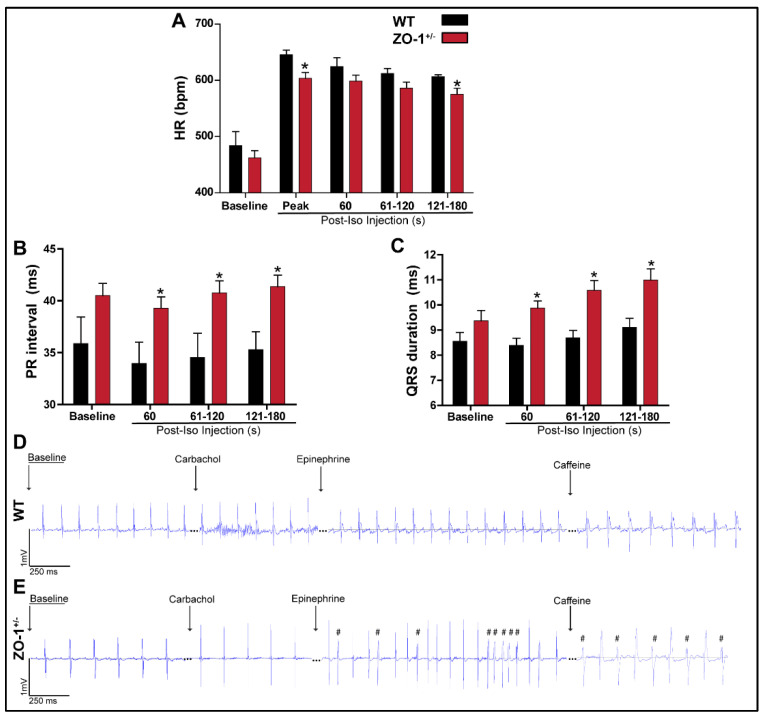
Electrocardiographic analysis of ZO-1^+/−^ mice. (**A**–**C**) Adult ZO-1^+/−^ mice showing a reduction in peak heart rate, prolonged PR interval and wider QRS duration compared to the WT control mice post ISO-stimulation. (* *p* < 0.05 for **A**–**C**) (**D**,**E**) Radio-implanted telemeter recordings of WT and ZO-1^+/−^ mice after intraperitoneal (IP) administration of carbachol (0.15 mg/kg) followed by 2 mg/kg of epinephrine and an IP caffeine dose of 120 mg/kg, ZO-1^+/−^ mice alone, display multiple PVCs and short runs of bigeminy (denoted by # in F) (WT, *n* = 3, two male and one female mice and ZO-1^+/−^, *n* = 3, male mice)).

ECG parameters were collected at baseline as well as in the presence of Isoproterenol (ISO) to mimic conditions of adrenergic stimulation utilizing subsurface ECG (Figure 4). Analysis of ECG parameters in ZO-1^+/−^ mice acquired in sinus rhythm (SR) revealed a significant reduction in peak heart rate, and a prolongation of the QRS and PR intervals in the presence of ISO (Figure 4A–C). Baseline arrhythmia was not present in either cohort. Additionally, we designed a dosing sequence to reproduce sudden adrenergic stimulation in the setting of parasympathetic drive. Here, radio-telemeters were surgically implanted in adult WT and ZO-1^+/−^ mice and baseline ECG data were collected which were consistent with previous subsurface ECG data. A cohort of WT and ZO-1^+/−^ animals were subject to a protocol consisting of carbachol (0.15 mg/kg) to mimic parasympathetic drive followed by application of 2 mg/kg epinephrine. An IP dose of caffeine (120 mg/kg) was administered to initiate a sudden spike in heart rate as well a raise in intracellular calcium levels to promote an arrhythmogenic substrate. Recordings from ZO-1^+/−^ animals demonstrate a high incidence of premature ventricular contractions (PVCs), short runs of bigeminy, and episodes of non-sustained ventricular tachycardia while WT animals remained in sinus rhythm indicative of a greater proclivity to arrhythmia in the absence of normal ZO-1 expression (Figure 4D,E). Taken together, ZO-1^+/−^ mice displayed electrical changes under adrenergic stimulation in the absence of structural phenotypes.

### 3.5. ZO-1^+/−^ Myocytes Display Prolonged Action Potential Duration and Spontaneous Diastolic Depolarization

**Figure 5:**
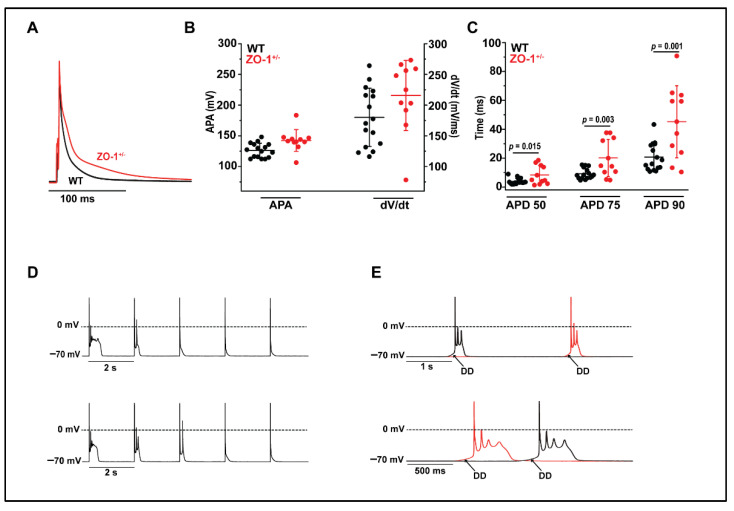
ZO-1^+/−^ ventricular myocytes (VMs) display prolonged action potential duration and spontaneous activity. (**A**) Representative AP recordings from WT and ZO-1^+/−^ VMs. (**B**,**C**) Action potential duration at 50, 75 and 90% repolarization are significantly prolonged in ZO-1^+/−^ myocytes while action potential amplitude (APA) and the maximum rate of depolarization (dV/dt) are unaffected. (**D**) Paced APs in ZO-1^+/−^ VMs display early after depolarizations (EADs) often triggering ectopic activity. (**E**) ZO-1^+/−^ VMs at rest display spontaneous diastolic depolarization (DD) leading to triggered activity (for (**B**,**C**), WT, *n* = 4; *n* = 16 and ZO-1^+/−^, *n* = 4; *n* = 11).

In ZO-1^+/−^ mice as compared to WT, it was hypothesized that some degree of dysregulation of EC and signal propagation occurred at the cellular level. This hypothesis was in part due to the known role of ZO-1 in the stabilization and regulation of connexin-43, in addition to evidence of indirect interactions with Na_V_1.5 at the cardiomyocyte perinexus [2,26]. To investigate this, we performed patch-clamp studies on single ventricular myocytes in ZO-1^+/−^ and WT mice. Action potential duration (APD) at 50, 75, and 90% repolarization were significantly longer in ZO-1^+/−^ ventricular myocytes compared to WT cells (3.85 ms +/− 2.08 vs 8.35 ms +/− 6.51; 9.20 ms +/− 3.75 vs 20.14 ms +/− 12.85; 20.71 ms +/− 9.58 vs. 45.18 ms +/− 24.96 respectively mean +/− S.D.) (Figure 5A,C).

Action potential amplitude (APA) and maximum rate of depolarization (dV/dt) were not statistically altered (Figure 5B). While arrhythmogenic early after depolarizations (EADs) were never observed in WT cells (Figure 5D), stimulation at 1 Hz frequently elicited EADs in ZO-1^+/−^ cells (Figure 5D). Furthermore, there were many instances of ZO-1^+/−^ cells displaying unsolicited (non-paced) diastolic depolarizations leading to spontaneous activity with multiple EADs (Figure 5E).

### 3.6. ZO-1 Haploinsufficiency Attenuates I_Na_ Current Density 

**Figure 6:**
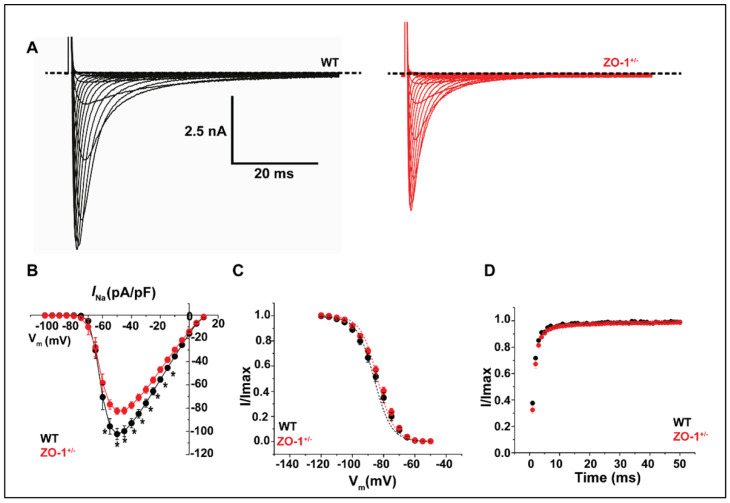
Reduced ZO-1 expression reduces Na_V_1.5 mediated current density (*I*_Na_). (**A**) Representative whole cell recordings of *I*_Na_ in WT and ZO-1^+/−^ ventricular myocytes. (**B**) ZO-1^+/−^ VMs display reduced current density, * *p* < 0.05 while (**C**) voltage dependent inactivation and (**D**) time dependent recovery are unaltered (for (**B**–**D**), WT, *n* = 3; *n* = 10 and ZO-1^+/−^, *n* = 3; *n* = 12).

Na_V_1.5 plays a major role in initiating the cardiac action potential and is localized primarily to the ID [27]. Voltage-clamp experiments were carried out in order to examine the effects of reduced ZO-1 expression on cardiac sodium current (*I*_Na_) density. *I*_Na_ recorded from ZO-1^+/−^ VMs displayed significantly lower current density (pA/pF), relative to WT VMs, at several experimental membrane voltages (Figure 6A,B).

**Figure 7:**
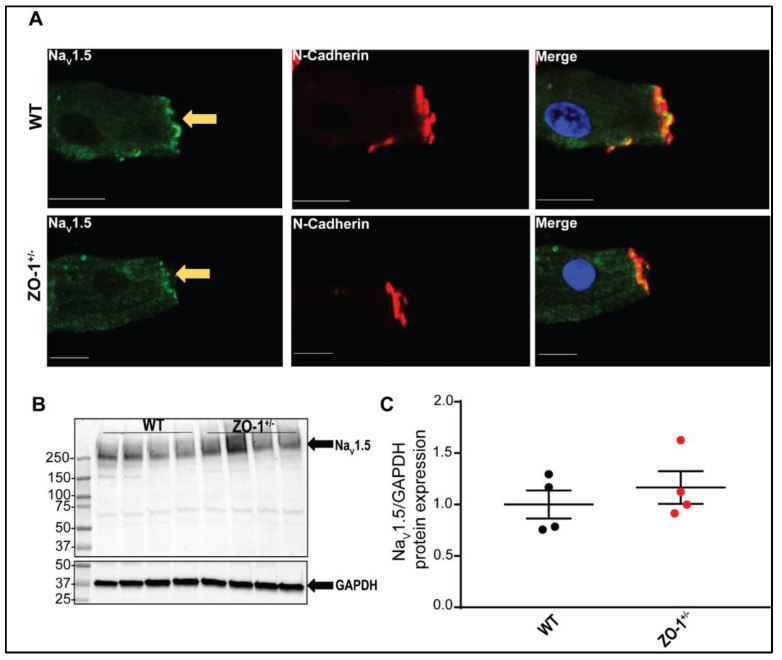
Na_V_1.5 expression and localization in ZO-1 deficiency. (**A**) Localization of Na_V_1.5 (green) and N-cadherin (red) in WT (*n* = 5, three male and two female mice) and ZO-1^+/−^ (*n* = 5, three male and two female mice) ventricular myocytes (scale bars = 20 μm; arrows indicate ID). DAPI (blue) denotes nuclei. Secondary antibodies were used as negative controls. (**B**,**C**) Immunoblotting and quantitative analysis of normalized Na_V_1.5 protein expression reveal no significant changes between WT and ZO-1^+/−^ heart lysates (*n* = 4 for each genotype, WT, two male and two female mice and ZO-1^+/−^, four male mice).

There were no significant effects on voltage-dependent inactivation and time-dependent recovery from inactivation (Figure 6C,D). Immunofluorescence labeling of Na_V_1.5 in ZO-1 haploinsufficient myocytes revealed a detectable attenuation of Na_V_1.5 signal at the ID compared to the control myocytes (Figure 7A).

However, immunoblotting of Na_V_1.5 protein expression in ZO-1 haploinsufficient lysates revealed no significant differences in normalized expression compared to the control lysates (Figure 7B,C), these data support a critical role of ZO-1 expression in the regulation of myocyte membrane excitability and Na_V_1.5 function.

### 3.7. Late Sodium Current in ZO-1 Deficient Mice Displays an Enhanced Sensitivity to Adrenergic Stimulation 

**Figure 8:**
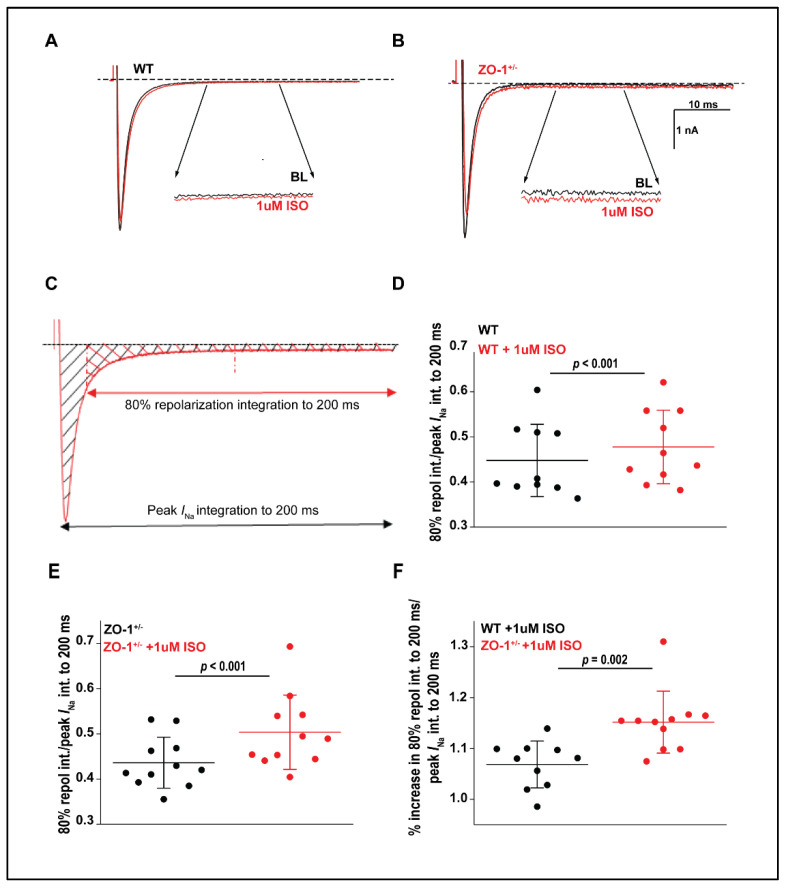
ZO-1 deficiency displays an enhanced sensitivity to adrenergic stimulation. (**A**,**B**) Representative whole recordings of *I*_Na_ before and after application of 1 µM isoproterenol (ISO). (**C**) Schematic presentation of the *I*_Na, L_ analysis. (**D**–**E**) Integration of *I*_Na, L_ adjusted to peak in WT and ZO-1^+/−^ myocytes displays a sensitivity to 1 µM ISO. (**F**) The percent increase in *I*_Na, L_ integration following exposure to 1 µM ISO is significantly larger in ZO-1^+/−^ VMs. Horizontal Bars represent mean and vertical bars represent SD (for (**D**–**F**), WT, *n* = 3 (two female and one male mice); *n* = 10 and ZO-1^+/−^, *n* = 3 (two female and one male mice; *n* = 11).

To assess the role of ZO-1 in the regulation of the arrhythmogenic late sodium current (*I*_Na,L_), WT and ZO-1 deficient myocytes were treated with isoproterenol (1 μM) to mimic increased adrenergic tone. The *I*_Na,L_ recordings from ZO-1 deficient myocytes displayed a significantly larger increase in late and persistent sodium current in the presence of 1 um ISO indicating a greater sensitivity to adrenergic stimulation (Figure 8A–F). Overall, these data support a role of ZO-1 in adrenergic-mediated increases in *I*_Na,L_.

### 3.8. ZO-1 Haploinsufficiency Displays an Increase in I_Ca_ Current Density

**Figure 9:**
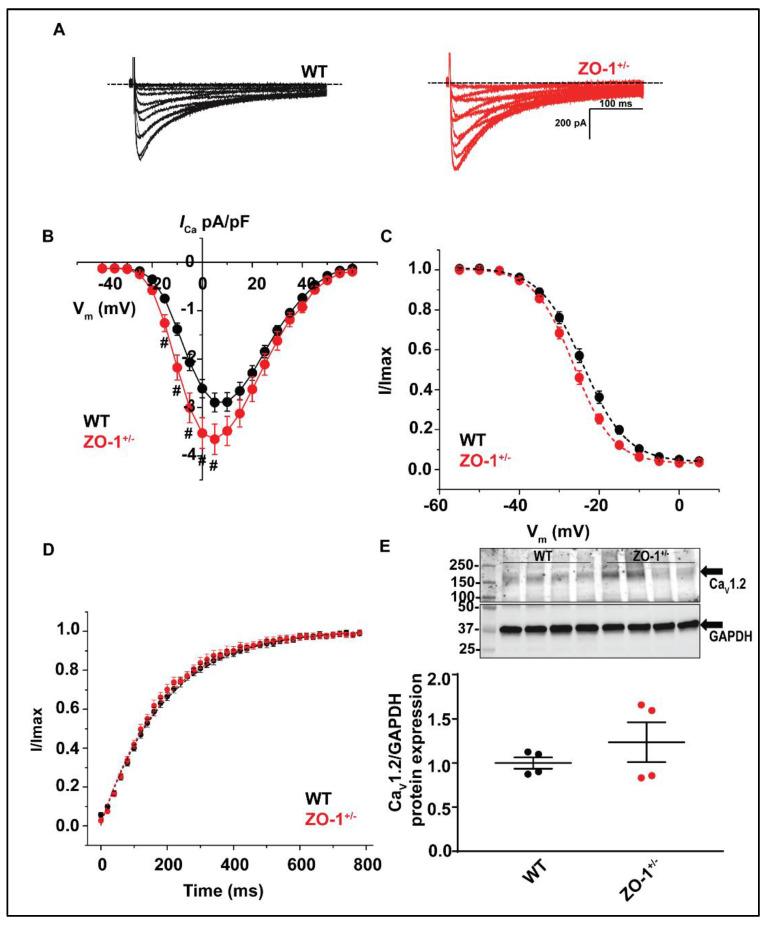
ZO-1^+/−^ myocytes display an increase in Ca_V_1.2 mediated current (I_Ca_). (**A**) Representative whole cell recordings of I_Ca_ in WT and ZO-1^+/−^ ventricular myocytes. (**B**) ZO-1^+/−^ myocytes display an upregulated current density at several membrane voltages, # *p* < 0.05 while (**C**) voltage dependent inactivation and (**D**) time dependent recovery remain unaffected (for (**B**), WT, *n* = 4 (four male mice); *n* = 12 and ZO-1*^+/−^*, *n* = 3 (two male and one female mice); *n* = 11. For (**C**,**D**), WT, *n* = 4 (four male mice); *n* = 11 and ZO-1^+/−^, *n* = 3 (two male and one female mice); *n* = 9). (**E**) Immunoblotting and quantitative analysis of normalized Ca_V_1.2 protein expression reveal no changes between WT and ZO-1^+/−^ heart lysates (*n* = 4 for each genotype), WT (two male and two female mice) and ZO-1^+/−^ (four male mice).

The observation of longer APDs and the frequent incidence of EADs and spontaneous activity led us to examine possible effects of reduced ZO-1 expression on Ca_V_1.2 mediated calcium current (*I*_Ca_). Whole cell patch clamp recordings from ZO-1^+/−^ VMs displayed a significantly larger *I*_Ca_ density than WT VMs at several experimental voltages relevant to the plateau phase of the action potential (Figure 9A,B).

Voltage dependent activation of *I*_Ca_ displayed a hyperpolarizing shift in V ½ that was nearing significance (*p* = 0.052), but time dependent recovery was unaffected (Figure 9C,D). Immunoblotting and quantitative analysis of Ca_V_1.2 protein expression revealed no significant changes in normalized protein expression in ZO-1 deficient lysates (Figure 9E). No changes were noted in Ca_V_1.2 localization between WT and ZO-1 deficient ventricular myocytes (Figure S2) Taken together, our patch-clamping recordings (Figure 9) suggest a role of ZO-1 in the regulation of Ca_V_1.2 mediated calcium current.

## 4. Discussion

Sudden cardiac death (SCD) accounts for 15–20% of total deaths in the world [28]. Defects in adhesion, cytoskeleton, and other ancillary non-ion channel proteins in cardiomyocytes can produce cardiac arrhythmia and SCD [29]. ZO-1 is known to play a role in the regulation of heart disease and several studies have reported the levels of ZO-1 in patients with end-stage heart failure. While Laing and colleagues reported a reduction in ZO-1 and Cx43 levels in the ID of patients with heart failure [1], Bruce et al. found an increase in ZO-1 levels associated with a significant decrease in Cx43 in the ventricular tissues of patients with end-stage heart failure [12,30]. Our expression data acquired from ventricular tissues of patients with diverse etiologies of heart failure (Figure 1) confirmed a significant reduction in ZO-1 levels in these heart lysates. Transcript analysis of *TJP1* revealed a trending downregulation of *TV1* and a significant downregulation of *TV3* in failing hearts with a notable upregulation of *TV4* suggesting a possible compensatory role for this isoform that has yet to be determined. However, expression of different transcript variants was not assessed at the protein level, which impacts the translational value of these findings.

ZO-1 is found within the ID at the adherens and gap junctions as established binding partners to N-cadherin and Cx43 respectively [31]. ZO-1 plays an integral role in the maintenance of GJs through regulation of the size of Cx43 aggregates forming functional GJs wherein the loss of ZO-1/Cx43 binding results in large Cx43 aggregates at the GJ [25]. The perinexus, a region immediately adjacent to GJs, is the proposed site where this regulatory relationship takes place [32,33]. ZO-1 was recently shown to form a complex with both connexin-45 and coxsackievirus, and adenovirus receptor (CAR) in AV nodal cells, which has been shown to regulate AV node function [15]. Additionally, tamoxifen-induced loss of ZO-1 in AV nodal cells was accompanied by a reduction in connexin-40 expression and a mislocalization in the ID of the AV node tissue [14]. These data convey the intricate role of ZO-1 in the form and function of cardiomyocytes through complex protein networks. However, limited data has been reported on the loss of ZO-1 in cardiac myocytes and the onset of ventricular arrhythmogenesis.

Here, we investigated the role of ZO-1 in cardiac disease, and in particular arrhythmia through biochemical, cellular, and physiological studies in a ZO-1 haploinsufficient murine model (Figure 2). Cardiac tissues from ZO-1^+/−^ mice showed dysregulation of both ID protein expression and localization, as well as gross changes in ID morphology (Figure 3 and Table S1). TEM studies demonstrated that ZO-1 deficiency altered the structure of gap junctional plaques, as was evident by the reduced step length and peak-to-peak amplitude of the discs themselves. Moreover, internal membranes of haploinsufficient mice showed an increase in T-tubule axial diameter compared to WT, and higher percentage of non-extended SR (Table S1 and Figure S1). Derangement of the T-tubule structure alone has been shown to make the myocardium more susceptible to arrhythmia, a result that is recapitulated in this model [34,35].

In many studies, it has been determined that arrhythmia can be driven by abnormal automaticity of myocardial tissue, however most arrhythmia is associated with a trigger that, in the presence of proper substrate, can become fatal [36,37]. Triggered activity in our study is represented in findings associated with adrenergic stimulation and calcium handling in the ZO-1 haploinsufficient mice. Based on our data, ZO-1^+/−^ mice showed conduction abnormalities after adrenergic stimulation and following caffeine induced calcium release (Figure 4) [38]. After application of intraperitoneal epinephrine, ZO-1^+/−^ mice demonstrated premature ventricular contractions (PVCs) and non-sustained ventricular tachycardia (NSVT). Application of caffeine resulted in bigeminy. Neither phenotype was elicited by similar treatments in WT animals. These discoveries point to our supposition that a loss of ZO-1 expression in myocardium can be a substrate for arrhythmia in the setting of physiologic stress. Notably, echocardiographic studies in our model did not reveal any alteration in cardiac function, which was previously reported by Zhang et al. in a conditional knockout model of ZO-1 in the heart [15]. However, limited reduction in cardiac function or ejection fraction without gross histological changes was noted by Dai et al. 20 d post tamoxifen injection in an inducible cardiomyocyte specific knockout model of *TJP1* [14].

At the ID, ZO-1 is central to the normal localization and function of Cx43 [39]. Specifically, in ZO-1 knockdown models, large Cx43 aggregates were mislocalized throughout the VM and were found to have lost an integral regulatory binding relationship at the perinexus where ZO-1 is responsible for regulating the size of GJs [31]. In our model, ZO-1^+/−^ mice confirm these findings in that immunofluorescent staining of Cx43 shows large aggregates of GJs throughout the VM. Moreover, TEM images revealed an increased distance between IDs at the GJ, further supporting dysregulation at this region and suggesting uncoupling with the loss of ZO-1 [40]. Further studies to elucidate possible changes to the gross propagation of conduction in ZO-1^+/−^ hearts through the use of cardiac mapping may be considered here. Additionally, our functional data in ZO-1^+/−^ myocytes revealed an isoproterenol-mediated increase in *I*_Na,L_ (Figure 8) and an increase in *I_Ca_* density (Figure 9) at rest, both of which may contribute to altered excitability and prolonged APD at phase 0 and phase 2, respectively, therefore resulting in conduction delay [41]. It is known that prolonged APD, through several studied mechanisms, can be a source for reentrant arrhythmias and triggered activity in myocardial cells [42,43]. In fact, while ZO-1^+/−^ VMs showed prolonged APDs, we also observed EADs, DADs, and spontaneous diastolic depolarizations with APA and dV/dt not statistically altered (Figure 5). Of note, DADs have been previously associated with conduction block when they are followed by impulses that produce supra-threshold triggered activity suggesting that DADs by themselves may result in conduction delay [44].

We did not investigate the role of NCX in our model; however, we do know that ZO-1^+/−^ myocytes demonstrate an increased *I_Ca_* density (Figure 9). Of note, the impact of ZO-1 deficiency on calcium currents may be an indirect result of compensation and not a direct regulation by ZO-1. While functional data revealed a possible mechanism for arrhythmia, it is still unclear as to why an isolated reduction in ZO-1 expression should result in reduced *I*_Na_ density and an increase in *I*_Ca_ density. Previous research has shown that cardiomyocyte specific loss of ZO-1 in mice results in qualitative loss of Na_V_1.5 expression in AV nodal cells, but not in ventricular cells. However, this study lacks functional experiments and current recordings. Additionally, the authors reported an increase in ZO-2 expression in the ventricular cells (not in AV nodal cells) and proposed a compensatory role of ZO-2 in the ventricle [15]. Moreover, only one study has demonstrated an indirect binding of Ca_v_1.2 and ZO-1, however, no mechanistic role was defined [45]. Furthermore, ZO-1 deficiency in our haploinsufficient mice did cause an isoproterenol-induced upregulation of the pro-arrhythmic late sodium current (Figure 8). Late sodium current increases in response to adrenergic stimulation and has previously been linked to fatal forms of congenital and acquired arrhythmic conditions [46,47,48,49]. These data support our finding that a decrease in ZO-1 expression results in myocardial substrate that is susceptible to abnormal cardiac conduction, that when triggered, can result in arrhythmia.

## 5. Limitations

In our study, a few limitations are important to address for further study. Most importantly, our mouse model was a total body ZO-1 haploinsufficient knockout. Repeating experiments addressed in this study in a cardiac-specific knockout mouse model would certainly be the next step in evaluating the mechanism of arrhythmia in the setting of reduced cardiac ZO-1 expression. Given EP results, and ECG findings, conduction mapping of whole murine hearts would be essential to further elucidating the mechanism of conduction delay and foci of arrhythmia in these mice.

## Data Availability

All data are contained within the article and Supporting Information document.

## Supplementary Materials

The following are available online at https://www.mdpi.com/article/10.3390/cells11040665/s1, Figure S1: TEM images in WT and ZO-1^+/−^ mice, Figure S2: Ca_V_1.2 localization in WT and ZO-1 deficient ventricular myocytes, Table S1: Summary of TEM findings in WT and ZO-1^+/−^ ventricular myocytes and Table S2: Summary of echocardiographic parameters in WT and ZO-1^+/−^ mice.
