# Supplementary material for: Altered Expression of Zonula occludens-1 Affects Cardiac Na+ Channels and Increases Susceptibility to Ventricular Arrhythmias
Source: Cells. 2022 Feb 14;11(4):665. doi: 10.3390/cells11040665 (PMC8870063; doi:10.3390/cells11040665)
Supplement: Supplementary file 1 [file cells-11-00665-s001.zip › cells-1568885-supplementary.pdf]

Supporting information

Altered expression of Zonula occludens-1 affects cardiac Na^+ channels and increases susceptibility to ventricular arrhythmias

Authors

Mona El Refaey[1,2 # *], Sara Coles[3 #], Hassan Musa[1,4], Tyler L. Stevens[1,4], Michael J. Wallace[1,4], Nathaniel P. Murphy[5], Steve Antwi-Boasiako[1], Lindsay J. Young[1], Heather Manring[6], Jerry Curran[1], Michael A. Makara[1], Kelli Sas[1], Mei Han[1], Sara N. Koenig[1,4], Michel Skaf[1], Crystal F. Kline[6], Paul ML Janssen[1,4], Federica Accornero[1,4], Maegen A. Borzok[7] and Peter J. Mohler[1,4,8]

Affiliations

[1] Frick Center for Heart Failure and Arrhythmia Research; The Dorothy M. Davis Heart and Lung Research Institute, The Ohio State University Wexner Medical Center, Columbus, OH, USA

[2] Department of Surgery, Division of Cardiac Surgery, The Ohio State University College of Medicine and Wexner Medical Center, Columbus, OH, USA

[3] Department of Medicine, Duke University School of Medicine, Durham, NC, USA

[4] Department of Physiology and Cell Biology, The Ohio State University College of Medicine and Wexner Medical Center, Columbus, OH, USA

[5] Department of Medicine, Northwestern Medicine, Chicago, IL, USA

[6] Comprehensive Cancer Center, The Ohio State University College of Medicine and Wexner Medical Center, Columbus, OH, USA

[7] Department of Natural Sciences, Mansfield University of Pennsylvania, Mansfield, PA, USA

[8] Department of Internal Medicine, Division of Cardiovascular Medicine, The Ohio State University College of Medicine and Wexner Medical Center, Columbus, OH, USA

[#] contributed equally

[*] Correspondence: Mona.elrefaey@osumc.edu ; Tel. 614-366-2748 (MER)

Supplemental Tables

Supplemental Table 1 (Table S1)

Supplemental Table 2 (Table S2)

Supplemental Figures

Supplemental Figure 1 (Figure S1)

Supplemental Figure 2 (Figure S2)

Table S1. Summary of TEM findings in WT and ZO-1$^{+/-}$ ventricular myocytes

	WT	ZO-1$^{+/-}$
**ID amplitude (mm)**	0.4 ± 0.03	0.3 ± 0.02*
**AJ membrane distances (nm)**	34.9 ± 1.0	33.3 ± 0.8
**DJ membrane distances (nm)**	31.9 ± 1.4	30.9 ± 1.1
**GJ membrane distances (nm)**	11.5 ± 0.8	14.6 ± 1.0*
**Tread width (mm)**	1.8 ± 0.1	1.6 ± 0.2
**Step length (mm)**	1.7 ± 0.1	1.2 ± 0.1*
**T-tubule axial diameter (mitochondrial-myofilament, nm)**	168.4 ± 6.9	191.0 ± 9.0
**T-tubule axial diameter (myofilament-myofilament, nm)**	64.3 ± 3.5	90.5 ± 4.9*
**Fully extended SR (%)**	72.3 ± 3.0	69.6 ± 4.2
**Partially extended SR (%)**	24.6 ± 3.0	17.9 ± 3.7
**Not extended SR (%)**	3.1 ± 1.4	12.5 ± 3.7*

n=3 (female mice) per genotype, *$p < 0.05$. ID: intercalated disc, SR: sarcoplasmic reticulum, AJ: adherens junctions, DJ: desmosome junctions, GJ: gap junctions

Table S2. Summary of echocardiographic parameters in WT and ZO-1$^{+/-}$ mice.

Gender	Genotype	IVSd (cm)	LVIDd (cm)	EDV (mL)	LVPWd (cm)	IVSs (cm)	LVIDs (cm)	ESV (mL)	EF (%)	FS (%)	LVPWs (cm)
Male	WT	0.06	0.41	0.17	0.08	0.08	0.32	0.08	52.28	22.73	0.09
Male	WT	0.05	0.38	0.13	0.07	0.09	0.27	0.05	62.28	28.7	0.1
Male	WT	0.05	0.375	0.135	0.04	0.06	0.295	0.065	50.205	21.58	0.06
Male	WT	0.066667	0.3566667	0.116667	0.05	0.1	0.2433333	0.04	66.17	31.3233	0.07666667
Female	WT	0.05	0.43	0.19	0.07	0.08	0.29	0.06	66.04	31.35	0.09
Female	WT	0.05	0.36	0.12	0.06	0.08	0.25	0.04	65.06	30.52	0.09
Female	WT	0.0575	0.335	0.0975	0.0475	0.0625	0.2425	0.0375	62.8425	29.065	0.0725
	Average	0.054881	0.3780952	0.137024	0.059642857	0.078929	0.2729762	0.053214	60.69679	27.89548	0.0827381
Female	ZO-1$^{+/-}$	0.05	0.32	0.09	0.07	0.08	0.22	0.03	65.22	30.53	0.08
Female	ZO-1$^{+/-}$	0.056667	0.3066667	0.076667	0.073333333	0.06	0.2133333	0.023333	66.5	31.38667	0.11
Male	ZO-1$^{+/-}$	0.066667	0.34	0.1	0.063333333	0.076667	0.25	0.043333	56.89667	25.33333	0.08333333
Male	ZO-1$^{+/-}$	0.053333	0.3333333	0.096667	0.053333333	0.063333	0.2566667	0.043333	53.24667	23.20333	0.06666667
Male	ZO-1$^{+/-}$	0.05	0.4	0.16	0.06	0.07	0.28	0.06	62.83	29.11	0.09
	Average	0.055333	0.34	0.104667	0.064	0.07	0.244	0.04	60.93867	27.91267	0.086
	p-values	0.9116	0.0843	0.1168	0.5581	0.2396	0.1174	0.168	0.8763	0.7551	0.7103

n=7 for WT and n=5 for ZO-1$^{+/-}$. IVSd: Interventricular septal end diastole, LVIDd: Left ventricular internal diameter end diastole, EDV: End-diastolic volume, LVPWd: Left ventricular posterior wall end diastole, IVSs: Interventricular septal end systole, LVIDs: Left ventricular internal diameter end systole, ESV: End-systolic volume, EF: ejection fraction, FS: fractional shortening, LVPWs: Left ventricular posterior wall end systole.

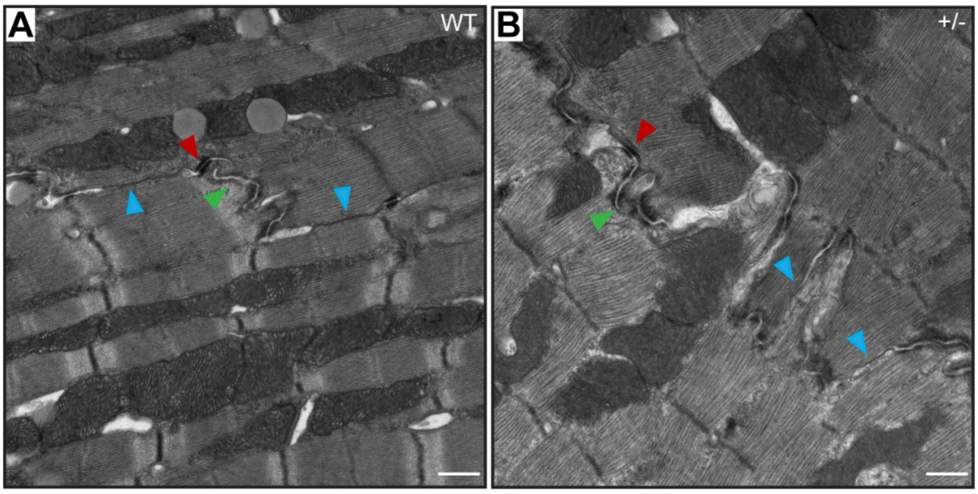

Figure S1. TEM images in WT and ZO-1$^{+/-}$ mice. TEM micrographs of intercalated disc between ventricular myocytes in WT (A) and ZO-1$^{+/-}$ (B) mice (n=3 (female mice) per genotype)**.** Red arrows denote desmosome Junctions (DJ), green arrows for adherens junctions (AJ) and blue arrows for gap junctions (GJ). Scale bars equal 500 nm.

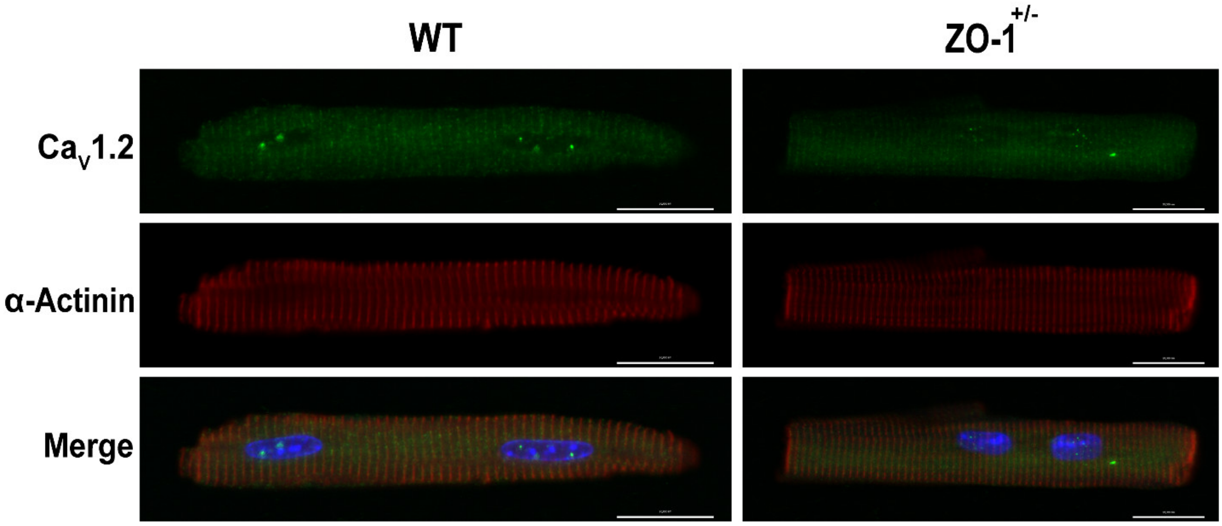

Figure S2. $Ca_V1.2$ localization in WT and ZO-1 deficient ventricular myocytes. Localization of $Ca_V1.2$ (green) and α-actinin (red) in WT and ZO-1$^{+/-}$ deficient myocytes. DAPI (blue) denotes nuclei. n=3/genotype (WT: 1 male and 2 female mice and ZO-1$^{+/-}$: 2 male and 1 female mice). Scale bars equal 20 μm.
